# Editorial: Advances and new perspectives in management of adult spine deformities of different aetiology

**DOI:** 10.3389/fsurg.2025.1666708

**Published:** 2025-08-13

**Authors:** L. Scaramuzzo, A. Gasbarrini, L. Proietti

**Affiliations:** ^1^ Department of Aging, Orthopaedics and Rheumatological Sciences, Fondazione Policlinico Universitario Agostino Gemelli IRCCS, Rome, Italy; ^2^ Department of Geriatrics and Orthopaedics, Sacred Heart Catholic University, Rome, Italy; ^3^ Department of Spine Surgery, IRCCS Istituto Ortopedico Rizzoli, Bologna, Italy; ^4^ Department of Biomedical and Neuromotor Sciences, Alma Mater Studiorum University of Bologna, Bologna, Italy

**Keywords:** spine deformities, scoliosis, hyperkyphosis, new technologies, surgical procedures

**Editorial on the Research Topic**
Advances and new perspectives in management of adult spine deformities of different aetiology

In recent years, the number of surgeries for adult spine deformities (ASDs) has continuously increased. The management of this kind of spine deformity is highly demanding and characterized by high costs. A continuous effort has to be made in the future to improve and standardize the treatment of this disease considering all the possible aetiologies. In order to reach these results, an improvement in predictability, safety, and sustainability is needed. Due to the extensive variability of ASD aetiologies and presentation and the many factors pertinent to patient outcomes, ASD is a very large field in which technological advances and new approaches could be successfully experimented with. The improvement of the treatment of this disease, considering its high impact on a patient's daily life, has to be centred not only on radiographic results and intraoperative technologies but the possibility of outcome prediction with machine learning algorithms has to be considered and implemented as well.

The aim of this Research Topic was to explore how the management of spinal deformities has evolved over time and identify the new perspectives and technological innovations in this field.

The first contribution of the volume by Fava et al. deals with a rare case of familial dysautonomia (FD), a severe congenital disease with a high incidence of kyphoscoliosis. The treatment and life expectancy of this pathology have improved, the incidence of diagnosis of kyphoscoliosis has increased, and nowadays, in specialized centres, the surgical treatment for deformities in rare diseases has become common. The use of new devices like silver-coated instrumentation and the use of new bioglasses allowed for surgical treatment in a severely compromised patient with a high risk of infection and a severe deformity in which all the conservative treatments had failed.

The second contribution by Romaniyanto et al. is a review of the literature on the advances of surgical treatment for deformities from spinal tuberculosis or Pott's disease. The conclusion reached by the authors underlines how the recent phase of surgical treatment of spine tuberculosis reflects a shift towards technology-driven approaches, including minimally invasive techniques, artificial intelligence, and machine learning, with the leadership of the orthopaedic surgeons in the field.

The third contribution by Peng et al. is centred on the improvement of the anaesthesiologic management of kyphotic deformities due to ankylosing spondylitis. These patients in the past were rarely surgically treated, and when surgery was performed, the risk of complications generally was higher than the benefits themselves. The authors, by describing an innovative and patient-centred anaesthesiologic and rehabilitative plan, show a possible way of optimizing the management of difficult airways and respiratory regulation, guiding circulation and fluid management through comprehensive monitoring. This protocol can reduce the factors that aggravate complications, improving the intra- and postoperative outcomes and the operability of these complicated patients.

The fourth contribution by Velluto et al. deals with the use of a new type of 3D-printed porous titanium cage compared with a traditional one in titanium in the minimally invasive treatment of degenerative deformities. The use of a minimally invasive lateral approach has, over the years, allowed for enlarging the surgical indication to older or compromised patients, reducing operative time and complications. The study analyses the fusion pattern of these new devices, comparing them with the traditional titanium cages, showing how the architecture of porous titanium cages offers a promising solution for increasing bone ingrowth and bridging space, and supporting successful spinal fusion while minimizing the risk of subsidence.

The fifth contribution by Wang et al. reports the possibility of treating a double post-traumatic deformity in a patient with ankylosing spondylitis with a closed reduction in a halo-vest and an open fixation of both cervical and lumbar injury. It is the first report of using a halo-vest to treat simultaneous double spine fractures–dislocation in a patient with AS, and it underlines the possibility of renewed use of consolidated devices like the halo-vest.

The sixth contribution by Zhou et al. evaluates the parameters that affect the postoperative clinical outcome of patients affected by degenerative deformities and explores the appropriate PI-LL range, considering the age and ethnicity of the patients. The authors, after a careful analysis of a large series of patients, conclude that the appropriate PI-LL is affected by PI and age. As the age increased, the LL needed to be restored could be reduced in order to improve the clinical outcome.

The central theme of this volume was fulfilled by these contributions, and we hope that future interest in applying new techniques and technological innovations to improve care standards in the treatment of spine deformities of different aetiologies could allow reduction of the risk of complications and provide pre-operative individualized counselling regarding optimal treatment approaches.

